# Evaluation of Polygenic Determinants of Non-Alcoholic Fatty Liver Disease (NAFLD) By a Candidate Genes Resequencing Strategy

**DOI:** 10.1038/s41598-018-21939-0

**Published:** 2018-02-27

**Authors:** Alessia Di Costanzo, Francesca Belardinilli, Diego Bailetti, Marialuisa Sponziello, Laura D’Erasmo, Licia Polimeni, Francesco Baratta, Daniele Pastori, Fabrizio Ceci, Anna Montali, Gabriella Girelli, Bruna De Masi, Antonio Angeloni, Giuseppe Giannini, Maria Del Ben, Francesco Angelico, Marcello Arca

**Affiliations:** 1 Departments of Internal Medicine and Medical Specialties, “Sapienza” University, Rome, Italy; 2 Molecular Medicine, “Sapienza” University, Rome, Italy; 3 Experimental Medicine, “Sapienza” University, Rome, Italy; 4 Anatomical, Histological, Forensic Medicine and Ortopedics Sciences, “Sapienza” University, Rome, Italy; 5 Cellular Biotechnologies and Hematology, “Sapienza” University, Rome, Italy; 6 Immunohematology and Transfusion Medicine Unit, “Sapienza” University, Rome, Italy; 7 Public Health and Infectious Diseases, “Sapienza” University, Rome, Italy

## Abstract

NAFLD is a polygenic condition but the individual and cumulative contribution of identified genes remains to be established. To get additional insight into the genetic architecture of NAFLD, GWAS-identified *GCKR, PPP1R3B, NCAN, LYPLAL1* and *TM6SF*2 genes were resequenced by next generation sequencing in a cohort of 218 NAFLD subjects and 227 controls, where *PNPLA3* rs738409 and *MBOAT7* rs641738 genotypes were also obtained. A total of 168 sequence variants were detected and 47 were annotated as functional. When all functional variants within each gene were considered, only those in *TM6SF2* accumulate in NAFLD subjects compared to controls (*P* = 0.04). Among individual variants, rs1260326 in *GCKR* and rs641738 in *MBOAT7* (recessive), rs58542926 in *TM6SF2* and rs738409 in *PNPLA3* (dominant) emerged as associated to NAFLD, with *PNPLA3* rs738409 being the strongest predictor (OR 3.12, 95% CI, 1.8-5.5, *P* < 0.001). A 4-SNPs weighted genetic risk score value >0.28 was associated with a 3-fold increased risk of NAFLD. Interestingly, rs61756425 in *PPP1R3B* and rs641738 in *MBOAT7* genes were predictors of NAFLD severity. Overall, *TM6SF2*, *GCKR*, *PNPLA3* and *MBOAT7* were confirmed to be associated with NAFLD and a score based on these genes was highly predictive of this condition. In addition, *PPP1R3B* and *MBOAT7* might influence NAFLD severity.

## Introduction

Non-alcoholic fatty liver disease (NAFLD), is a multifactorial disease characterized by an increased hepatic triglyceride content (>5.5% of liver weight) in the absence of an excess of alcohol consumption, HVC infection, familial hypobetalipoproteinemia or endocrine disorders^1,2^. NAFLD, which currently represents the leading cause of liver damage in developed countries^3^, has well established risk factors such as insulin resistance associated with overweight, physical inactivity and type 2 diabetes mellitus (T2DM)^4^. However, epidemiological, familial and twin studies have clearly indicated that the risk of NAFLD has also a strong genetic component^5^.

In the last few years, a large number of genetic investigations, employing single candidate gene as well as genome-wide association studies (GWAS) strategies, have provided compelling evidence that several gene variants are associated with NAFLD^5^. In particular, the rs738409 C > G change in the Patatin-like Phospholipase domain-containing 3 (*PNPLA3*) gene, coding for the I148M protein variation, has been identified as a major determinant of inter-individual and ethnicity-related differences in hepatic fat content^6,7^. The mechanism by which this substitution induces liver fat is related to an impaired hepatocellular triglycerides hydrolysis and increased lipogenesis associated to the 148 M allele^8,9^. More recently, the Transmembrane 6 Superfamily Member 2 (*TM6SF2*) E167K variant has also been shown to increase NAFLD susceptibility^10^. This effect appears to be due to an impaired mobilization of neutral lipids for very low-density lipoprotein (VLDL) assembly and secretion by the liver in E167K carriers^11–13^.

Furthermore, GWAS studies have indicated additional *loci* whose involvement in the pathogenesis of liver steatosis is less established. In particular, Speliotes E.K. *et al*.^14^ reported that variants in Protein Phosphatase 1 Regulatory Subunit 3B (*PPP1R3B*), Glucokinase Regulatory Protein (*GCKR*), Neurocan (*NCAN*) and Lysophospholipase Like 1 (*LYPLAL1*) genes were associated to the presence of NAFLD. Finally, based on results obtained in patients with alcoholic liver disease (ALD)^15^, Mancina *et al*.^16^ demonstrated an association between the rs641738 in the membrane Bound O-Acyltransferase Domain Containing 7 gene (*MBOAT7*) and the occurrence and progression of NAFLD. The minor allele (T) was significantly associated with high liver fat content only in European Americans, but not in African Americans and Hispanics. Moreover, this variant showed an additive effect with *PNPLA3* and *TM6SF2* single nucleotide polymorphisms (SNPs) in determining the risk of liver fibrosis^17^. Overall, these findings clearly indicate that the genetic predisposition to NAFLD results from a combination of several variants, which may influence different steps of hepatic lipid and carbohydrate metabolism^18^.

Despite this wealth of knowledge, the proportion of genetic risk of NAFLD explained by the identified *loci* remains modest (<5%). This might be because the majority of GWAS tag SNPs are common and/or lie in intergenic or intronic regions^19^. Moreover, GWAS did not capture rare or low frequency risk variants with moderate/strong effects, which could explain a part of this missing heritability. An effective way to overcome these limitations is to re-sequence the entire coding portion of candidate genes to capture all non-genotyped risk alleles. This strategy has already been successfully employed for various conditions where subjects with well distinct phenotypes were genotyped^20^. In addition, the candidate genes resequencing strategy, by providing a comprehensive evaluation of the polygenic architecture of NAFLD, would allow to weighing the overall as well as the individual contribution of different variants to the risk of this complex trait.

Here we provide the evaluation of genetic determinants of NAFLD using the sequencing analysis of candidate genes emerged from GWAS. To this aim, we have re-sequenced the coding regions of *GCKR, PPP1R3B, LYPLAL1, NCAN* and *TM6SF2* genes in NAFLD and control subjects, where *PNPLA3* rs738409 and *MBOAT7* rs641738 genotypes were also obtained.

The association of individual variants with NAFLD has been evaluated by using logistic regression analysis. Furthermore, following the logic of recent studies that have tested in complex disorders the combined impact of multiple genetic variants^21^, we determined a polygenic score for NAFLD based on identified risk alleles.

## Results

### Subjects characteristics

Baseline characteristics of study participants are reported in Table 1. Compared with controls, NAFLD subjects were older, showed higher indices of adiposity and increased plasma triglycerides (TG) and reduced HDL-C concentrations (all *P* < 0.001). Also fasting plasma levels of glucose, insulin and HOMA_IR_ values were significantly higher in NAFLD compared with control subjects (all *P* < 0.001). As expected, the prevalence of T2DM and metabolic syndrome (MetS) was higher in NAFLD than in controls. Moreover, subjects with NAFLD were more frequently smokers and hypertensives (*P* < 0.001). A statistically significant elevation of ALT (*P* < 0.001), AST (*P* = 0.008) and γGT (*P* < 0.001) were seen in NAFLD compared with control subjects. Among NAFLD subjects, 164 (76.3%) were classified as having moderate to severe liver steatosis according to Hamaguchi’s criteria.

**Table 1:** Clinical and metabolic characteristics of study subjects.

	NAFLD	Controls	*P*
N	218	227	
Age *(years*)	54 (46–60)	49.7 (41–58)	<**0.001**
Gender*(M/F)(%)*	69.7/30.3	63.0/37.0	**ns**
BMI *(kg/m*^*2*^*)*	29.2 (26.5–32.5)	24.9(23.2–27.1)	<**0.001**
Waist circumference, *(cm)*	106.1 ± 13.5	91.2 ± 9.91	<**0.001**
Smokers, n (%)	66 (30.6)	44 (19.7)	**0.043**
T2DM, n (%) *	54 (24.8)	3 (1.3)	<**0.001**
MetS, n (%)	126 (57.8)	18 (7.9)	<**0.001**
Statins, n (%)	50 (22.9)	28 (12.3)	**0.003**
Systolic BP (mmHg)	130 (120–140)	120 (110–135)	<**0.001**
Diastolic BP (mmHg)	80 (80–85)	80 (70–80)	<**0.001**
TC *(mg/dl)*	199.8 ± 41.1	201.0 ± 38.2	**ns**
TG *(mg/dl)*	134 (99–183)	87 (68–116)	<**0.001**
HDL-C *(mg/dl)*	44 (37.5–54)	58 (49–68)	<**0.001**
LDL-C *(mg/dl)*	125.3 ± 60.2	121.7 ± 33.9	**ns**
Fasting Blood Glucose *(mg/dl)*	95 (85–112)	85 (77–90)	<**0.001**
Fasting Insulin *(UI/L)*	12.7 (8.9–19.9)	5.7 (4.0–7.8)	<**0.001**
HOMA_IR_	3.0 (2.1–5.0)	1.14(0.77–1.65)	<**0.001**
ALT *(UI/I)*	27 (19–42)	15 (12–20)	<**0.001**
AST *(UI/I)*	23 (19–30)	21 (18–26)	**0.008**
γ-GT (UI/I)	27 (17–41)	19 (15–26)	<**0.001**

Data are expressed as percentage, mean (±SD) and median (25^th^–75^th^ percentile range) as appropriate.

Abbreviations: AST, aspartate aminotransferase; ALT, alanine aminotransferase; BMI, body mass index; BP, blood pressure; y-GT, gamma glutamyl transferase; HDL, high density lipoprotein; HOMA-IR, homeostasis model of insulin resistance (fasting plasma glucose in mg/dL x fasting insulin in U/L)/405; LDL, low density lipoprotein; MetS, metabolic syndrome (defined by the NCEP-ATP III Expert Panel criteria^43^); NAFLD, non-alcoholic fatty liver disease; TC, total cholesterol; T2DM, type 2 diabetes mellitus.

### DNA re-sequencing

Overall, 168 variants were identified, of which 100 were intronic and 68 exonic. Among exonic variants, 43 were nonsynonymous (NS), 2 nonsense, 2 frameshift and 21 synonymous (Supplementary Table S1). Thirty one (65.9%) were classifiable as rare (MAF < 0.01), 5 (10.6%) as low frequency/less common (0.01 ≤ MAF < 0.05) and 3 (6.4%) common variants (MAF ≥ 0.05). Six exonic variants (12.7%) were not been previously reported in *dbSNP* and thus submitted to *EXAC database* (http://exac.broadinstitute.org). Forty-seven variants were annotated as functional (nonsense, frameshift and nonsynonymous), 23% in *GCKR*, 14.8% in *LYPLAL1*, 34% in *NCAN*, 8.5% in *PPP1R3B* and 19.4% in *TM6SF2* genes. These variants were considered for further analyses.

The list of identified variants with their in *silico* prediction of deleteriousness is reported in the Supplementary Table S2.

### Enrichment of gene variants in NAFLD and controls

**Figure 1:**
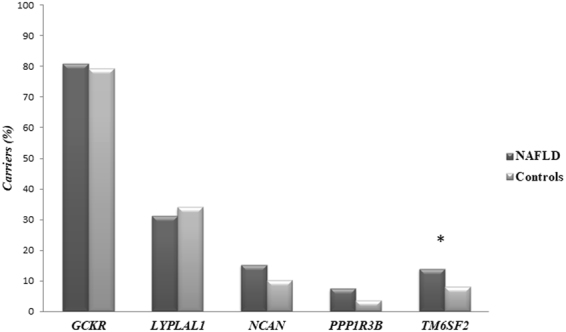
Enrichment of gene variants in NAFLD and controls. Percentage of subjects carrying at least one functional variant. In each group (NAFLD cases and controls) we count the number of subjects positive for at least one functional variant within each gene. *χ^2^ = 4.14, *P* = 0.04. *Odd Ratio unadjusted: OR = 2.0, 95% CI, 1.0-4.0, *P* = 0.04. In the model were included all subjects observed as carrying at least one functional variant per gene in NAFLD patients *vs*. controls.

Figure 1 shows the percentage of subjects carrying at least one functional variant within each gene in study groups. Overall, 80% of subjects with NAFLD were positive for at least one variant in *GCKR*, 31% for *LYPLAL1*, 15% for *NCAN*, 8% for *PPP1R3B* and 14% for *TM6SF2* genes. Among controls, 79% were positive for at least one variant in *GCKR*, 34% in *LYPLAL1*, 10% in *NCAN*, 4% in *PPP1R3B* and 8% in *TM6SF2* genes. Although variants in *NCAN* and *PPP1R3B* appeared to be more frequent in cases compared with controls, only those in *TM6SF2* reached the statistical significance (OR = 2.0, 95% CI, 1.0-4.0, *P* = 0.04). However, after correction for multiple comparisons, the association of *TM6SF2* gene was no longer significant.

### Association of individual variants with NAFLD

In order to investigate the effect of genetic variants on NAFLD susceptibility, each identified sequence variation was included in a stepwise regression analysis. As all study subjects were also genotyped for the *PNPLA3* rs738409 and *MBOAT7* rs641738, these variants were also considered. As reported in Table 2, rs1260326 C/T in *GCKR*, rs58542926 C/T in *TM6SF2*, rs738409 C/G in *PNPLA3* and *MBOAT7* rs641738 T allele emerged as significantly associated with the presence of NAFLD (all *P* ≤ 0.05).

**Table 2:** Genotype frequencies and Odds Ratios (ORs) of variants associated with NAFLD.

Gene	SNP ID	NAFLD (N/%)	Controls (N/%)	Genotype model					
		N = 218	N = 227	χ^2^	*P*-value	OR (95% CI)	Unadjusted *P-value*	OR (95% CI)	Adjusted* *P-value*
***PNPLA3***	**rs738409**								
	CC	92 (42.2)	123 (54.2)						
	CG	91 (41.7)	56 (24.7)			2.2 (1.41–3.33)	<0.001	2.5 (1.30–4.85)	0.006
	GG	35 (16.1)	48 (21.1)	14.6	0.001	0.9 (0.58–1.62)	0.9	4.2 (2.07–8.85)	<0.001
*Dominant Model*	CG + GG	126 (57.8)	104 (42.8)	6.39	0.014	1.6 (1.11–2.35)	0.012	3.2 (1.79–5.59)	<0.001
*Recessive Model*	GG	35 (16.1)	48 (21.1)	1.89	0.168	0.7 (0.44–1.15)	0.16	3.0 (1.55–5.93)	0.001
***GCKR***	**rs1260326**								
	CC	43 (19.7)	49 (21.6)						
	CT	90 (41.3)	123 (54.2)			0.8 (0.51–1.36)	0.46	0.8 (0.44–1.74)	0.69
	TT	85 (39.0)	55 (24.2)	11.7	0.003	1.8 (1.03–2.99)	0.04	1.8 (0.86–3.73)	0.11
*Dominant Model*	CT + TT	175 (80.3)	178 (78.8)	0.23	0.62	1.1 (0.70–1.77)	0.62	1.2 (0.62–2.24)	0.61
*Recessive Model*	TT	85 (39.0)	55 (24.2)	11.2	0.001	2.0 (1.32–3.00)	0.001	1.9 (1.12–3.46)	0.018
***TM6SF2***	**rs58542926**								
	CC	161 (88.0)	214 (94.3)						
	CT	25 (11.5)	11 (4.8)			2.5 (1.20–5.25)	0.014	2.4 (0.91–6.65)	0.07
	TT	—	2 (0.9)	8.349	0.015	—			
*Dominant Model*	CT + TT	25 (11.5)	13 (5.7)	4.693	0.041	2.1 (1.06–4.28)	0.033	2.2 (0.84–5.77)	0.10
***MBOAT7***	**rs641738**								
	CC	61 (28.0)	78 (34.4)						
	CT	105 (48.2)	111 (48.9)			1.21 (0.78–1.85)	0.38	0.90 (0.49–1.65)	0.74
	TT	52 (23.9)	38 (16.7)	4.243	0.120	1.75 (1.02–2.99)	0.04	1.75 (0.82–3.71)	0.14
*Dominant Model*	CT + TT	157 (72.0)	149 (65.6)	2.107	0.147	1.34 (0.90–2.02)	0.14	1.09 (0.62–1.92)	0.74
*Recessive Model*	TT	52 (23.9)	38 (16.7)	3.487	0.062	1.55 (0.97–2.48)	0.06	1.86 (0.96–3.59)	0.06

Data express the absolute numbers and percentages of cases and controls. Only variants showing a *P* value ≤ 0.06 were reported in the table.

*Models were adjusted by Age (years), Gender (M/F), BMI (kg/m^2^), HOMA_IR_, TG (mg/dl) and genotypes were considered as dominant or recessive (Logistic Regression analysis, Enter Method).

Abbreviations: BMI, Body Mass Index, *GCKR*, glucokinase (hexokinase 4) regulator gene, HOMA_IR_, homeostasis model of insulin resistance, *MBOAT7*, membrane-bound O-acyltransferase domain-containing 7 gene, *NAFLD, non-alcoholic fatty liver disease. PNPLA3*, Patatin-like phospholipase domain-containing protein 3 gene, *TM6SF2*, Transmembrane 6 superfamily Member 2 gene, TG, Triglycerides, OR, odd ratio, 95% CI, 95% CI confidence interval.

After adjustment for covariates such as age, gender, body mass index (BMI), HOMA_IR_ and TG, a dominant model of inheritance best explained the association with NAFLD of rs738409 C/G *PNPLA3* (OR = 3.2, 95% CI, 1.79-5.59, *P*_adj_ < 0.001). Conversely, the association of rs1260326 C/T *GCKR* (OR = 1.9, 95% CI, 1.12-3.46, *P*_adj_ = 0.018) fitted better with a recessive model of inheritance. A similar trend was observed for *TM6SF2* rs58542926 T and *MBOAT7* rs641738 T alleles, although with a borderline level of significance. However, it must be noted that a real estimation of the effect of *TM6SF2* variant in the dominant or recessive model could not be provided because of the low frequency of the T-allele (167 K) (only two homozygous subjects). Thus, all calculations were based on the dominant model of inheritance.

When these 4 NAFLD-associated variants were tested together, they explained, overall, about 7% of the genetic risk of NAFLD and the rs738409 in *PNPLA3* ranked as the strongest predictor (OR = 3.12, 95% CI, 1.8-5.5, *P* < 0.001) when adjusted for conventional risk factors (Table 3). Hosmer-Lomeshow goodness of fit test showed that the model combining both genetic and non-genetic variables explained the observed data (X^2^_6_ = 16.45; P = 0.036) with a predictive ability of 58.2%. The results did not change even after including or excluding from the model plasma TG and glucose levels. It is worth to mention that when in this model HOMA_IR_ was removed from covariates, *TM6SF2* T allele reemerged as a significant predictor of NAFLD (OR = 3.6, 95% CI, 1.40-9.27, *P*_adj_ = 0.008). This might be explained by the observation that carriers of this variant showed higher levels of HOMA_IR_ compared to non-carriers (3.2 (2.1-6.0) *vs*. 1.43 (0.88-1.93), *P* = 0.002, respectively).

**Table 3:** Independent associations of genetic variants with NAFLD.

Gene, SNP ID	*β*	OR (95% CI)	*P* -value	*P_*adj*_*-value*
*PNPLA3, rs738409* *Dominant Model*	1.14	3.12 (1.8–5.5)	<0.001	0.001
*GCKR*, rs1260326 *Recessive Model*	0.64	1.90 (1.1–3.4)	0.028	0.039
Age (years) BMI (kg/m^2^) HOMA_IR_ TG (mg/dl)	0.02 0.19 0.71 0.01	1.02 (1.00–1.05) 1.21 (1.11–1.31) 2.03 (1.56–2.65) 1.01 (1.00–1.01)	0.040 <0.001 0.001 0.001	0.039 0.001 0.010 0.005

Stepwise regression analysis (Forward-Wald Statistic) were used to test the association of clinical and genetic factors with NAFLD. In the model were included: Age (years), Gender (M/F), BMI (kg/m^2^), HOMA_IR_, TG (mg/dl), rs738409 *PNPLA3* (dominant model), rs1230326 *GCKR* (recessive model), rs58542926 *TM6SF2* (dominant model) and rs641738 *MBOAT7* (recessive model). Only significant variables were reported.

*P was adjusted for multiple comparisons by using the bootstrap method.

Abbrevations: BMI, Body Mass Index, *GCKR*, glucokinase (hexokinase 4) regulator gene, HOMA_IR_, homeostasis model of insulin resistance, *PNPLA3*, Patatin-like phospholipase domain-containing protein 3 gene, TG, Triglycerides, OR, odd ratio, 95% CI, 95% CI confidence interval.

Next, we examined the proportion of risk of NAFLD conferred by gene-gene and gene-environment interaction. Although we did not identify any gene-gene synergy, *PNPLA3* rs738409 showed a significant inverse interaction with TG (OR = 0.9, 95% CI, 0.97-0.99, *P* = 0.0021) and HOMA_IR_ (OR = 0.33, 95% CI, 0.17-0.62, *P* = 0.001) but not with age, gender or BMI. Moreover, we observed a barely significant interaction between *TM6SF2* polymorphism and BMI (OR = 0.8, 95% CI, 0.63-1.01, *P* = 0.07). Conversely, no interactions between *GCKR* rs1260326 or *MBOAT7* rs641738 and conventional NAFLD risk factors were observed.

### Association of genetic variants with metabolic traits

After stratifying the study population by *TM6SF2* rs58542926 (dominant model), *GCKR* rs1260326 (recessive model), *PNPLA3* rs738409 (dominant model) and *MBOAT7* rs641738 (recessive model) genotypes, difference in clinical, anthropometric and biochemical indices were found across groups. NAFLD individuals carrying the minor T allele of rs58542926 (167 K) (N = 25) showed lower plasma total cholesterol (TC) (*P*_adj_ = 0.021) and TG (*P*_adj_ = 0.002) and higher AST (*P*_adj_ = 0.006) and ALT (*P*_adj_ = 0.012) levels when compared with NAFLD patients with wild-type C allele (N = 193). More importantly, the association with TC and TG levels was unchanged after adjustment for BMI, T2DM or statin therapy (all *P*_adj_ < 0.05). Similarly, NAFLD patients carrying CG or GG *PNPLA3* genotypes (N = 126) compared with non-carriers (N = 92) showed, lower BMI (*P*_adj_ = 0.001), lower TG levels (*P*_adj_ = 0.001) and HOMA_IR_ (*P*_adj_ = 0.004) and higher AST levels (*P*_adj_ < 0.001). Notably, the association of [CG + GG] genotypes with TG levels persisted even after adjustment for BMI and diabetes (*P*_adj_ = 0.01). On the contrary, no differences in clinical, anthropometric and biochemical indices were found across the *GCKR* rs1260326 or the *MBOAT7* rs641738 genotypes in patients with NAFLD.

### Genetic risk score (GRS) and the risk of NAFLD

**Figure 2:**
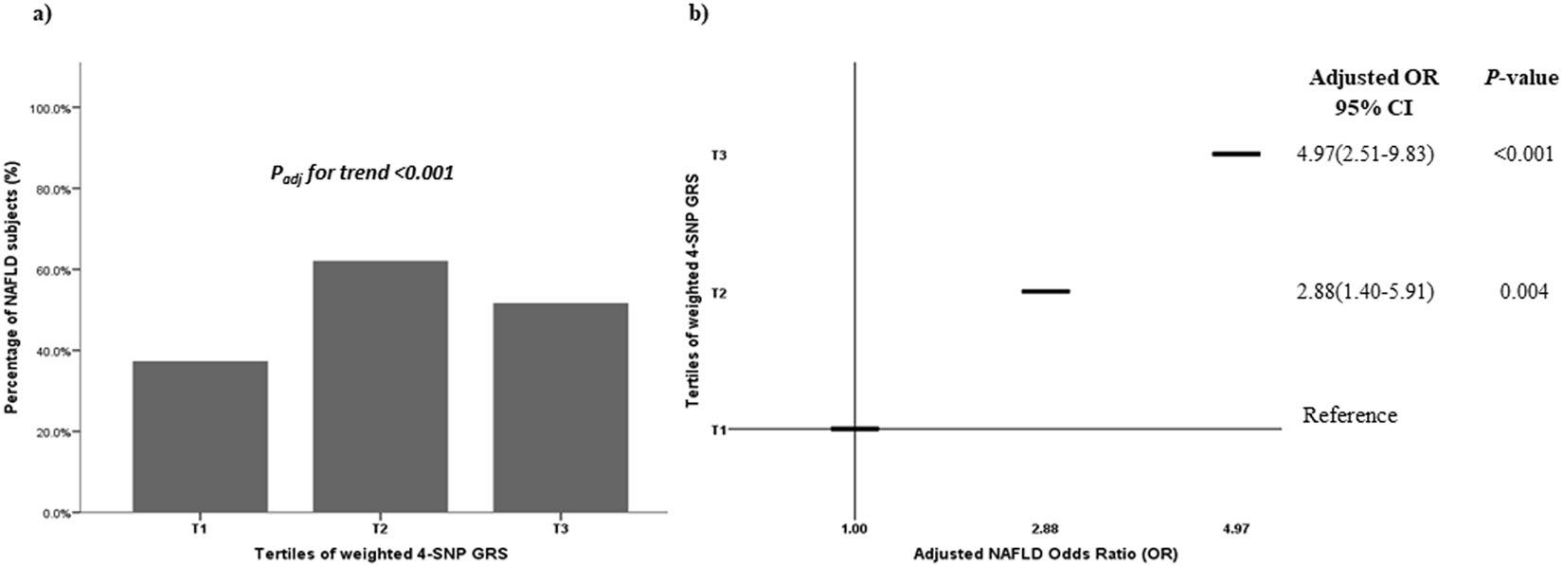
Association of weighted GRS with the risk of NAFLD. (**a**) Distribution of tertiles of weighted 4-SNP GRS in NAFLD patients; (**b**) NAFLD Odds Ratio (OR) adjusted for age, gender, BMI, HOMA_IR_ and TG across tertiles of weighted 4-SNP GRS. The weighted 4-SNP GRS was calculated by multiplying the sum of the number of risk alleles (0–2) with the corresponding effect sizes per allele as obtained from the Dallas Heart Study^22^. Tertiles boundaries were defined as follow: T1 GRS ≤0.1775; T2 GRS >0.1775 and ≤0.3877; T3 GRS >0.3887. (**a**) *P*_adj_ for trend. In the model were included age (years), gender (M/F), BMI (kg/m^2^), HOMA_IR_ and TG (mg/dl) and tertiles of weighted 4-SNSP GRS (χ^2^ Pearson followed by Stepwise Regression analysis). (**b**) Adjusted NAFLD OR. In the model were included age (years), gender (M/F), BMI (kg/m^2^), HOMA_IR_ and TG (mg/dl) and tertiles of weighted GRS (Stepwise Regression analysis, Forward-Wald Statistic).

The median values of weighted and unweighted 4-SNP GRS were significantly higher in NAFLD than in controls (median unweighted GRS: 3 (2–4) *vs*. 2 (2–3), *P* = 0.001; median weighted GRS: 0.38 (0.17–0.50) *vs*. 0.18 (0.12–0.44), *P* = 0.03, respectively). When weighted 4-SNP GRS values was distributed according to tertiles (Fig. 2, Panel *a*), the prevalence of NAFLD significantly increased along with increasing tertiles (χ2 = 14.9, *P* = 1 × 10^-4^) and the risk was significantly higher for GRS values above >0.28 (corresponding to the 2^th^ tertile) (Fig. 2, Panel *b*). This trend persisted even after adjustment for age, gender, BMI, HOMA_IR_ and TG levels.

### Association of genetic variants with severity of NAFLD

The association of genetic variants with the ultrasound-defined severity of NAFLD is reported in Table 4. A NGS-identified variant in *PPP1R3B* gene (rs61756425 G/T p.S41R) emerged as more frequent in NAFLD patients than in controls (χ^2^ = 16.11, *P* < 0.001) and as the strongest independent genetic predictor of severe hepatic steatosis (OR = 32.6, 95% CI, 4.22-251.4, *P*_adj_ = 0.001). This association was maintained even after bootstrap correction (two-tailed *P*_adj_ = 0.001). Similarly, the rs641738 in *MBOAT7* gene showed a significant effect on NAFLD severity (OR = 2.6, 95% CI, 1.10-6.28, *P*_adj_ = 0.022). As expected, age, BMI and HOMA_IR_ were detected as the non-genetic significant predictors of severity of NAFLD.

**Table 4:** Predictors of NAFLD severity in the whole cohort.

*NAFLD degree*	*Variables*	*β*	OR (95% CI)	*P*-value	*P_*adj*_-value
*Severe*	*PPP1R3B, rs61756425* *MBOAT7, rs641738* Gender (F) BMI (kg/m^2^) HOMA_IR_	3.48 0.96 1.26 0.23 0.60	32.6 (4.22–251.4) 2.6 (1.10–6.28) 0.3 (0.11–0.70) 1.2 (1.15–1.38) 1.8 (1.51–2.22)	0.001 0.029 0.006 <0.001 <0.001	0.001 0.022 0.027 0.001 0.001

Stepwise regression analysis (Forward-Wald Statistic) were used to test the association of clinical and genetic factors with NAFLD. In the model were included: Age (years), Gender (M/F), BMI (kg/m^2^), HOMA_IR_, TG (mg/dl), rs738409 *PNPLA3* (dominant model), rs1230326 *GCKR* (recessive model), rs58542926 *TM6SF2* (dominant model) and rs641738 *MBOAT7* (recessive model). Only significant variables were reported.

*P was adjusted for multiple comparisons by using the bootstrap method.

Abbrevations: BMI, Body Mass Index, HOMA_IR_, homeostasis model of insulin resistance, *MBOAT7*, membrane-bound O-acyltransferase domain-containing 7 gene, NAFLD, Non-alcoholic fatty liver disease, TG, Triglycerides, OR, odd ratio, *PPP1R3B*, Protein Phosphatase 1 Regulatory Subunit 3B, 95% CI, 95% CI confidence interval.

## Discussion

NAFLD is a complex trait whose genetic component has been explored by many studies using different approaches^18^. Although several genes and genetic variants have been identified as involved in the occurrence of the disease, not all were consistently confirmed. Moreover, their combined effect on NAFLD susceptibility has rarely been explored.

In agreement with previous studies^6,7,10,14,16,18,22,23^, we found that *PNPLA3* rs738409, *GCKR* rs1260326, *TM6SF2* rs58542926 and *MBOAT7* rs641738, but not the other GWAS identified variants^14^, were genetic contributors to NAFLD in our cohort.

Hepatic fat accumulation results from an imbalance between TGs acquisition, synthesis, utilization and secretion^24^ and, as yet described, the *PNPLA3* I148M, *TM6SF2* E167K and *GCKR* P446L polymorphisms promote steatosis through interaction with distinct metabolic mechanisms. Both genotypes in *PNPLA3* and *TM6SF2* influence the ability to export very low-density lipoproteins (VLDLs) from the liver^8–13,17^. In addition, p.P446L is a loss-of-function variant that results in increased phosphorylation of glucose^25^, glycolysis and fatty acid synthesis^26^. Similarly, recent findings indicated that the rs641738 in *MBOAT7* gene, by decreasing the expression of MBOAT7 enzyme, unfavorably affects the acyl remodeling of phospholipid acyl-chain in the liver^16^. Taken together, these data indicate that a biologically plausible mechanism by which these gene variants directly influence the development of NAFLD exists. On the other hand, they further support the notion that the impaired lipid handling by hepatocytes has a major causal role in the pathogenesis of NAFLD.

When considered on individual basis, *PNPLA3* variant ranked as the strongest genetic predictor of NAFLD followed by *GCKR*. In contrast, a weaker association was detected for *TM6SF2* and *MBOAT7*. These observations could be partially explained by the low frequency of *TM6SF2* risk allele as compared to *PNPLA3* risk allele^27^. In addition, the finding that NAFLD carriers of the *TM6SF2* 167K allele have increased levels of HOMA_IR_ might have masked the effect of this variant on NAFLD risk due to the predominant role of insulin resistance in the pathogenesis of NAFLD^28,29^. The finding on *MBOAT7* is more difficult to be interpreted. However, it must be pointed out that this gene has not been consistently associated with NAFLD in all ethnic groups^16^ and, when a role was demonstrated, the *MBOAT7* rs641738 variant showed the smallest effect in predisposing to fatty liver^22^.

Another interesting aspect of our findings is that, in agreement with previous studies, they do not fully support the notion that common variants are the major contributors to NAFLD susceptibility^20,30^. In fact, in our cohort the rs58542926 T allele (MAF ~ 7%) displayed a 2.5-fold risk of hepatic steatosis higher than the 1.9 fold risk associated to *GCKR* T allele (MAF ~ 42%). Thus, according to the hypothesis of Manolio T.A. *et al*.^20^, the lower frequency of rs58542926 in the general population could be the reason why we observed a larger effect of *TM6SF2* T allele differently to *GCKR* TT genotype in the occurrence of NAFLD. Moreover, it is important to consider that in our cohort 55% of rs1260326 TT carriers were also heterozygous or homozygous carriers for the *PNPLA3* rs738409, thus suggesting that the association with *GCKR* might be due to genetic bias.

Nevertheless, we confirmed that these genetic variants might act in an additive fashion. In fact, by using a 4-SNP GRS, we observed that the full combination of risk alleles increased the probability of hepatic steatosis up to 5 fold and this effect was present even after adjustment for traditional risk factors. These results emphasize the importance to consider a multiplicity of potentially involved gene variants when studying the genetic epidemiology of a common complex trait as NAFLD^31,32^. Of note, only four previous studies, carried out in different ethnic groups, have considered the combined effect of different genetic variants in determining fatty liver^17,22,33,34^. However, these Authors only performed genotyping tests for some of all previously GWAS-identified SNPs without exploring the entire coding region of NAFLD-associated *loci*. In addition, Krawczyk M. *et al*.^17^ concentrated their attention in evaluating the effect of the number of risk alleles (unweighted GRS) only on the grade of steatosis and fibrosis. Although our results need to be further evaluated in larger populations, they highlight the possibility to identify individuals at high risk of NAFLD by genotyping these genetic risk factors. Notably, EASL–EASD–EASO Clinical Practice Guidelines^35^ already suggest genotyping for *TM6SF2* and *PNPLA3* to select patients with higher risk of hepatic steatosis.

Our results indicated that the model including genetic and non-genetic variables accounts for the 58.2% of NAFLD heritability. These findings further confirm that the hepatic steatosis is a dynamic process that results from a constant interplay between genetic and environmental determinants and its heritability is not only due to the primary effect of *PNPLA3*, *TM6SF2*, *GCKR* and *MBOAT7* genotypes but also by the secondary effects of non-genetic factors. In contrast with Stender S. *et al*.^36^, we did not find any synergy between adiposity and genotypes. The lack of interaction with BMI could be related to the fact that we did not quantify the intrahepatic triglyceride content (IHTG) but we considered hepatic steatosis as binary outcome variable in a case-control study^37^. Thus, while we described an inverse interaction effect between *PNPLA3* genotypes HOMA_IR_ and TG in predicting the higher risk of hepatic steatosis, the modest sample size could be the reason why we did not identified synergy with BMI^37^.

Finally, we found a significant association between the *PPP1R3B* rs61756425 and *MBOAT7* rs641738 variants in predicting the severity of hepatic steatosis. Although *MBOAT7* has been previously associated with progression of NAFLD^16,17,38^, the observation on *PPP1R3B* is novel. This variant has never been identified in GWAS nor associated to severity of NAFLD. This is probably due to its very low frequency in the population, thus supporting the notion that the re-sequencing of entire coding region of candidate genes may capture non-genotyped low-frequency risk alleles^20,39^. It must be, however, pointed out that the high value with a wide 95% CI of OR associating *PPP1R3B* rs61756425 with NAFLD severity may suggest a low level of accuracy of this estimate, mainly due to the very low number of carriers of this variant. Therefore, additional evaluations in much larger cohorts are needed. On the other hand, very recent observations have challenged the role of *PPP1R3B* as genetic factor predisposing to liver fat accumulation. In fact, Stender S. *et al*.^40^ have shown in mice that the lack of *PPP1R3B* was associated with reduced glycogen and unchanged fat liver content; conversely, the hepatic overexpression of *PPP1R3B* caused accumulation of hepatic glycogen and elevated ALT levels without affecting triglycerides accumulation. Based on this evidence, the role of *PPP1R3B* rs61756425 variant in liver disease surely requires additional evaluation with a direct confirmation of liver histological changes associated with this variant.

Strengths and limitations of our study must be acknowledged. To the best of ours knowledge, this is the first study reporting a comprehensive evaluation of sequence variants detectable in the entire coding regions of NAFLD-associated *loci*. This allowed evaluating the individual as well as the cumulative contribution of identified variants to the risk of NAFLD. However, it must be recognized that the definition of NAFLD was based on ultrasound and not on direct measurement of hepatic fat content with a more accurate methods such as the liver magnetic resonance. In addition, the size of the sequencing sample was modest so that some relevant rare variant might have been missed. To this regard, it is noteworthy that the power analysis indicated that our experimental design was able to identify low frequency variants and to detect moderate effects size. More, we did not re-sequenced the *PNPLA*3 and the *MBOAT7* genes. For the former, we have considered the 148 M variant as it represents the only variant in this gene associated to hepatic fat content^41^. In fact, Donati B *et al*. by re-sequencing a cohort of children with early-onset histological NAFLD did not find any additional predictive rare variant in the *PNPLA3* gene^41^. In addition, we genotyped only the *MBOAT7* rs641738 variant as it was reported to display the major role in NAFLD^16,42^. Finally, an additional limitation of our study was the lack of replication of identified rare variants in an independent sample. However, it must be considered that in the present study we have re-sequenced well-known genetic determinants of NAFLD not requiring replication. Nevertheless, the analysis of rare variants was adjusted for multiple testing.

In conclusion, we confirmed the role of *PNPLA3*, *TM6SF2*, *GCKR* and *MBOAT7* gene variants as genetic determinants of NAFLD and we suggested a weighted GRS based on their additive and combined effect. We believe these results point the way towards a future feasibility of creating comprehensive risk factor panels, in which applying genetic testing for the individual-level NAFLD-risk prediction. If definitely confirmed, our GRS score could offer the opportunity to exclude low-risk patients from screening tests.

## Material and Methods

### Study subjects

We studied 445 Caucasian, unrelated subjects, 218 with echographically defined NAFLD and 227 healthy controls. The enrollment criteria as well as the protocol for clinical and biochemical characterization of study subjects have been reported elsewhere^43^. In brief, NAFLD subjects, were considered eligible after exclusion of secondary causes of liver steatosis such as previous viral infection, past or present history of alcohol abuse (defined as an average daily consumption >20 g/day), use of drugs known to influence the development of hepatic steatosis as well as clinical and biochemical evidence of chronic liver diseases. Healthy controls, recruited from blood donors, were selected based on the absence of advanced liver disease at ultrasound^43^. Liver ultrasonography was performed with a GE Vivid S6 apparatus equipped with a 3.5-MHz convex-array probe. All examinations were done by the same hepatologist and steatosis was assessed semi-quantitatively on a scale of 0–6: 0, absent; 1, 2 mild; 3, 4 moderate; and 5, 6 severe according to the Hamaguchi criteria^44^.

The study protocol was reviewed and approved by the Ethics Committee of Sapienza University of Rome, Policlinico Umberto I (Rome, Italy). Written informed consent was obtained from all participants in accordance with the principles of the Helsinki Declaration. All methods were carried out in accordance with the relevant guidelines and regulations.

### DNA analysis

#### Selection of candidate genes

The genes considered for next generation sequencing (NGS) were the following: *GCKR, NCAN, PPP1R3B, LYPLAL1* and *TM6SF2*. They were selected because reported by GWAS to be associated with NAFLD above a significance threshold of *P* < 10^−4^ for any tagging SNPs^10,14^. In our screening, we have also considered the *PNPLA3* rs738409 and the *MBOAT7* rs641738, as previously demonstrated to be genetic determinants of NAFLD^6,16^. The genotyping of these latter variants was performed in duplicate by TaqMan 5′-Nucleotidase assay having a concordance rate of 100%.

#### Next-generation sequencing (NGS)

A custom panel was designed with the help of the AmpliSeq designer online tool (https://www.ampliseq.com), which was employed to generate optimized primer designs for the five genes present in the human reference genome (hg19). The overall coverage of the design region was 99,9%. (Pipeline version 4.2). Amplicon library preparation was performed with the Ion Ampliseq Library kit v2.0 using 10 ng of DNA (Thermo Fisher Scientific). PCR products were partially digested using FuPa reagent, followed by the ligation of barcoded sequencing adapters (Ion Xpress Barcode Adapters kit; Life Technologies, Carlsbad, CA, USA). Final libraries were purified using Agencourt AMPure XP magnetic beads (Beckman Coulter, Brea, CA, USA) and quantified using a Qubit 3.0 Fluorometer (Thermo Fisher Scientific, Wilmington, DE). The individual libraries were diluted to a final concentration of 100 pM and were pooled and processed to library amplification using Ion PGM Template OT2 400 kit. Unenriched libraries were quality-controlled using Ion Sphere quality control measurement on a Qubit instrument. Following library enrichment (Ion OneTouch ES), libraries were processed for sequencing by using the Ion PGM Hi-Q Sequencing Kit v2.

#### Data filtering and analysis

Sequencing runs were analyzed using the Torrent Suite v4.4.3 analysis. SNPs and insertion/deletions were identified across the targeted subset of the reference genome (hg19) using the analysis plug-in Torrent Variant Caller with the parameter settings optimized for germline low stringency and minimal false positive calls. The output variant call format (VCF) file was then annotated through Ion Reporter (Ion Reporter™ Software 4.6) and wANNOVAR softwares (http://wannovar.wglab.org). All sequencing variants were filtered using our custom NGS pipeline. All variants with Depth Coverage (DP) ≥30, Genotype Quality (GQ) ≥30, Allele Frequency (AF) ≥33 and ≤50 or ≥70 and ≤100, with balanced Alternate Allele Observations on the forward strand (SAF) and Alternate Allele Observations on the reverse strand (SAR) were considered as high confident variants and used for further analysis. Twenty four variants in 77 subjects with moderate quality were retested by Sanger sequencing on an ABI PRISM 3130 XL Genetic Analyzer following standard protocols. Overall, 13 variants with AF <33 and DP <20 were not confirmed.

The damaging effect of identified missense variants were evaluated by *in silico* prediction softwares. SIFT, PolyPhen-2, Provean, SNP&GO and Mutation T@ster Prediction softwares were used. A collective predictive score, ranging from 0–10, was calculated as the sum of individual scores of the 5 tools utilized, each being 0 (Neutral/benign/Polymorphism) or 1 (possibly damaging by Polyphen) or 2 (Disease Causing/Probably Damaging). Variants were defined as damaging if reported as deleterious in at least three of five prediction tools.

All common (MAF >5%), low frequency and rare variants (MAF ≥1 or ≤5% and <1%, respectively) annotated as functional (nonsense, frameshift, splice-region and missense) were considered for the analysis.

#### Power Calculation

Power analysis was performed by the Genetic Association Study (GAS) Power Calculator (© 2017 Jennifer Li Johnson | University of Michigan), commonly used to compute statistical power for one-stage genetic association studies within the setting of additive or multiplicative genetic models. The prevalence of NAFLD in our population was estimated as 0.30 and the odd ratio (OR) for each risk allele of the tested genetic variants was set at approximately 2.0, as estimated in previous studies^45^. Assuming an allele frequency of 0.3 (for common variants) and 0.03 (for low-frequency variants) in the general population and an additive model for disease risk, with a sample size of 218 cases and 227 controls the expected power under a significance level of 0.05 was of 100% to identify common genetic associations and 87% to identify low-frequency genetic associations. Notably our analysis had a power of 0.80 to detect genetic effects with OR of at least 1.9 for low-frequency and 1.35 for common variants.

#### Genetic Risk Score Computation

The Genetic Risk Score (GRS) was calculated based on the four SNPs reaching the highest levels of significance for NAFLD: rs1260326 C/T in *GCKR*, rs58542926 C/T in *TM6SF2*, rs738409 C/G in *PNPLA3* and rs641738 C/T in *MBOAT7* genes. Two methods were used to create the GRS: a simple count method (unweighted GRS) and a weighted method (weighted GRS)^31,32^. The count method assumed that each SNP contributed equally to NAFLD risk and was calculated applying a linear weighting of 0, 1 and 2 to genotypes containing 0, 1, or 2 risk alleles, respectively. While in our population we found only two homozygous subjects for rs58542926 in *TM6SF2* gene this produced a score between 0 and 7, representing the maximum total number of risk alleles. The weighted 4-SNP GRS was calculated by multiplying each *β* -coefficient for the NAFLD phenotype obtained from the Dallas Heart Study^6,22^ by the number of corresponding risk alleles (0, 1, or 2) and then summing the products. The *β*–coefficient considered for each SNP were: 0.2653 (rs738409 *PNPLA3*), 0.2711 (rs58542926 *TM6SF2*), 0.0649 (rs1260326 *GCKR*) and 0.0575 (rs641738 *MBOAT7*). The 4-SNP GRS was modelled as a continuous variable and then categorized into tertiles.

#### Statistical analysis

The two-sample *t*-test (for parametric variables) or the Mann–Whitney test (for non-parametric variables) was used to compare the difference between case and control groups for quantitative traits, while Pearson’s χ^2^ test was used to compare discrete traits. Deviations of genotype frequency from the Hardy–Weinberg assumption were assessed using a χ^2^ test. Differences in allele and genotype frequencies between cases and controls were assessed by χ^2^ test under either dominant or recessive model of penetrance.

The enrichment of gene variants was evaluated by counting NAFLD cases and controls positive for at least on functional variant in each candidate gene.

Logistic regression analysis (Forward-Wald Statistic or Enter method) were adopted to assess the most significant model of inheritance for each SNP, the joint effects of genes and clinical variables and to evaluate gene-gene and gene-environmental interactions. The adequacy of the final model was assessed using Hosmer-Lameshow goodness-of-fit test. Furthermore, the Nagelkerke R^2^ was calculated to indicate how useful the explanatory variables in the model were in predicting NAFLD^46^. We further analysed the association of *PNPLA3* rs738409, *TM6SF2* rs58542926, *GCKR* rs1260326 and *MBOAT7* rs641738 with biochemical indices by using General Linear Model test with bootstrap correction including age and sex as covariates. TG and TC were also adjusted for BMI, diabetes and statin therapy while HOMA_IR_ was adjusted for BMI. For variables with skewed distributions (ALT, AST, TG, HOMA_IR_), a logarithm was applied before analysis to ensure that the residuals were approximately normal and had constant variance.

The effect of studied variants as well as of additional risk factors on the degree of hepatic steatosis were analyzed in logistic regression analysis (Forward-Wald Statistic) by comparing patients with severe NAFLD as having Hamaguchi score = 5–6 (N = 71) with patients without or with mild-moderate hepatic steatosis (Hamaguchi score = 0–4) (N = 362).

Associations between NAFLD risk and 4-SNP GRS were tested using Pearson correlations or logistic regression analysis.

Multiple comparisons were adjusted by bootstrap correction based on 1000 bootstrap samples with the aim to adjust raw p-value thus obtaining more robust estimates of standard errors and confidence intervals of parameters included in the models. Statistical significance was taken at nominal *P-*value < 0.05 for all comparisons. All analyses were performed using SPSS package (version 22.0) (SPSS, Inc., Chicago, IL, USA).

### Data availability statement

All data generated or analyzed during this study are included in this published article (and its Supplementary Information files). The datasets generated during and/or analyzed during the current study are not publicly available due to the lack of a specific patients’ consent but are made available by corresponding author based on reasonable request.

## Electronic supplementary material

Supplementary Table S1 and TableS2
