# Supplementary material for: Evaluation of Polygenic Determinants of Non-Alcoholic Fatty Liver Disease (NAFLD) By a Candidate Genes Resequencing Strategy
Source: Sci Rep. 2018 Feb 27;8:3702. doi: 10.1038/s41598-018-21939-0 (PMC5829219; doi:10.1038/s41598-018-21939-0)
Supplement: Supplementary file 1 — Supplementary Table S1 and TableS2 [file 41598_2018_21939_MOESM1_ESM.pdf]

Supplementary Material

EVALUATION OF POLYGENIC DETERMINANTS OF NON-ALCOHOLIC FATTY LIVER DISEASE (NAFLD) BY A CANDIDATE GENES RESEQUENCING STRATEGY.

Di Costanzo Alessia[1 *], Belardinilli Francesca[2], Bailetti Diego[3], Sponziello Marialuisa[1], D'Erasmo Laura[1], Polimeni Licia[1], Baratta Francesco[1, 4], Pastori Daniele[1, 4], Ceci Fabrizio[5], Montali Anna[1], Girelli Gabriella[6], De Masi Bruna[6], Angeloni Antonio[2], Giannini Giuseppe[2], Del Ben Maria[1], Angelico Francesco[7], Arca Marcello[1]

Supplementary Table S1. Number of exonic variants identified within candidate genes according to predicted effect.

	Exonic Variants	Non-synonymous	Nonsense	Frameshift	Synonymous
GCKR	14	8	2	1	3
LYPLAL1	9	6	-	1	2
PPP1R3B	5	4	-	-	1
NCAN	25	16	-	-	9
TM6SF2	13	9	-	-	4

GCKR, glucokinase (hexokinase 4) regulator gene, *LYPLAL1,* Lysophospholipase Like 1gene, *PPP1R3B*, Protein Phosphatase 1 Regulatory Subunit 3B gene, *NCAN*, Neurocan gene, *TM6SF2*, Transmembrane 6 superfamily Member 2 gene.

Supplementary Table S2. List of identified variants in candidate genes with evaluation of functional effect in NAFLD cases and controls**.**

Genes	Chr Position	dbSNP ID	Type	Protein change	MAF	NAFLD (N)	Controls (N)	Polyphen	SIFT	Provean	SNP & GO	Mut. Taster	Prob. Damaging
**GCKR**	chr2:27720483	exac	missense	p.Gln91Lys	0.00008	1	0	0	0	0	0	0	
-	chr2:27721130	exac	frameshift	p.Leu101AlafsTer22	0.00002	0	1	na	na	na	na	na	yes
-	chr2:27729720	novel	missense	p.Asp345Val	-	0	1	2	2	2	2	2	yes
-	chr2:27731054	novel	missense	p.Phe453Cys	-	1	0	2	0	0	0	2	
-	chr2:27730940	rs1260326	missense	p.Leu446Pro	0.4108	175	178	0	0	0	0	0	
-	chr2:27728609	rs138410297	missense	p.Arg259Gly	0.0005	0	1	2	2	2	0	0	yes
-	chr2:27745372	rs146053779	nonsense	p.Arg540Ter	0.0011	0	1	na	na	na	na	na	yes
-	chr2:27726437	rs147073127	missense	p.Gln234Pro	0.0033	1	3	0	2	0	0	0	
-	chr2:27730107	rs201754753	nonsense	p.Arg358Ter/ p.(R358*)	0.0002	1	0	na	na	na	na	na	yes
-	chr2:27746196	rs34792470	missense	p.His590Tyr	0.001	1	1	0	0	0	0	0	
-	chr2:27745373	rs8179249	missense	p.Arg540Gln	0.006	2	2	0	0	0	0	0	
**LYPLAL1**	chr1:219384894	exac	frameshift	p.Leu181PhefsTer13	0.00005	1	0	na	na	na	na	na	yes
**-**	chr1:219384896	exac	missense	p.Glu180Asp	0	1	0	0	0	0	0	2	yes
-	chr1:219366554	novel	missense	p.Glu108Lys	-	0	1	2	2	2	2	2	yes
-	chr1:219385003	rs146719365	missense	p.Thr216Ser	0.0005	1	0	0	0	0	0	0	
-	chr1:219384945	rs34201999	missense	p.Leu197Val	0.00006	0	1	1	2	0	0	2	yes
-	chr1:219383905	rs940570	missense	p.Ile131Met	0.01	1	1	0	0	0	0	0	
-	chr1:219383865	rs940571	missense/ near splice	n.321-7T>C/ p.Phe68Leu	0.01	65	75	na	na	na	na	na	yes
**NCAN**	chr19:19335209	novel	missense	p.Tyr249His	-	1	0	2	2	2	2	2	yes
-	chr19:19338200	exac	missense	p.Glu591Lys	0.0001	0	1	0	0	0	0	0	
**-**	chr19:19338650	novel	missense	p.Thr741Pro	-	1	0	1	2	0	0	0	
-	chr19:19338747	novel	missense	p.Ala773Val	-	2	0	0	2	0	0	0	

Genes	Chr Position	dbSNP ID	Type	Protein change	MAF	NAFLD (N)	Controls (N)	Polyphen	SIFT	Provean	SNP & GO	Mut. Taster	Prob. Damaging
**NCAN**	chr19:19359574	novel	missense	p.Asn1235Asp	-	0	1	1	0	0	0	2	
**-**	chr19:19338942	rs10426537	missense	p.Ser838Asn	0.0008	0	1	1	0	0	0	0	
-	chr19:19334995	rs112606625	missense	p.Arg214His	0.00003	0	1	0	0	0	0	2	
-	chr19:19356209	rs139427012	missense	p.Arg1194Cys	0.00002	1	1	2	2	2	0	0	yes
-	chr19:19338227	rs143497000	missense	p.Pro600Ala	0.00009	1	0	0	2	0	0	0	
-	chr19:19337630	rs149801377	missense	p.Val470Leu	0.00001	0	1	0	2	0	0	0	
-	chr19:19329924	rs2228603	missense	p.Pro92Ser	0.08214	25	18	1	0	0	0	0	
**-**	chr19:19339337	rs367609638	missense	p.Glu970Lys	0.00006	1	0	0	0	0	0	0	
-	chr19:19349218	rs372689346	missense	p.Asn1136Thr	0.00005	0	1	2	2	2	0	0	yes
-	chr19:19329873	rs373921518	missense	p.Arg75Trp	0.00006	1	0	2	2	2	2	2	yes
-	chr19:19349163	rs375854561	missense	p.Arg1118Cys	0.00016	1	0	2	2	2	0	2	yes
**-**	chr19:19349187	rs568608129	missense	p.Val1126Ile	0.00005	1	0	0	0	0	0	0	
**PPP1R3B**	chr8:8998354	rs201747156	missense	p.Pro270Ser	0.00001	1	2	0	0	0	0	0	
-	chr8:8999019	rs3748140	missense	p.Gly48Glu	0.0109	7	4	2	2	2	0	2	yes
-	chr8:8999039	rs61756425	missense	p.Ser41Arg	0.013	9	3	0	0	0	0	0	
-	chr8:8998959	exac	missense	p.Asn68Ser	0.00001	1	0	0	0	0	0	0	
**TM6SF2**	chr19:19381932	novel	missense	p.Pro33Leu	-	1	0	2	2	2	0	2	yes
-	chr19:19381028	rs117846705	missense	p.Val119Ile	0.0004	1	0	0	0	0	0	0	
-	chr19:19381881	rs184254301	missense	p.Ala50Val	0.002	1	0	1	0	0	0	0	
-	chr19:19378859	rs186811910	missense	p.Pro216Leu	0.0004	0	1	2	2	2	0	2	yes
-	chr19:19380513	rs187429064	missense	p.Leu156Pro	0.01	2	2	2	2	2	0	2	yes
-	chr19:19375583	rs188787025	missense	p.Leu342Met	0.001	0	1	1	0	0	na	2	
-	chr19:19381885	rs200492531	missense	p.Val49Leu	0.002	0	2	0	0	0	0	0	
-	chr19:19380514	rs368135661	missense	p.Leu156Val	0.00008	0	1	0	0	0	0	2	
-	chr19:19379549	rs58542926	missense	p.Glu167Lys	0.08	25	13	2	0	0	0	0	

Summary carrier counts and in *silico* prediction of functional effects are shown. Six exonic variants were not been previously reported in dbSNP and thus submitted to EXAC database (http://exac.broadinstitute.org).

MAF: Minor Allele Frequency evaluated by Exome Aggregation Consortium (ExAC) browser in European (Non-Finnish) population (http://exac.broadinstitute.org).

Probably Damaging. Variants were defined as Probably Damaging if reported as deleterious in at least three of five prediction tools. Frameshift, nonsense and splice region variants were considered as Probably Damaging.

GCKR, glucokinase (hexokinase 4) regulator gene, *LYPLAL1*, Lysophospholipase Like 1gene, *PPP1R3B*, Protein Phosphatase 1 Regulatory Subunit 3B gene, *NCAN*, Neurocan gene, *TM6SF2*, Transmembrane 6 superfamily Member 2 gene.
